# The Efficacy and Safety of Entecavir and Interferon Combination Therapy for Chronic Hepatitis B Virus Infection: A Meta-Analysis

**DOI:** 10.1371/journal.pone.0132219

**Published:** 2015-07-30

**Authors:** Qiao-Ling Xie, Ying Zhu, Ling-Hong Wu, Lin-Lin Fu, Yan Xiang

**Affiliations:** Department of Infectious Diseases. The First Affiliated Hospital of Dalian Medical University, Dalian, Liaoning, China; National Taiwan University Hospital, TAIWAN

## Abstract

The objective of this study was to evaluate the effectiveness and safety of entecavir (ETV) and interferon (IFN) combination therapy in the treatment of chronic hepatitis B (CHB) mono-infection via a meta-analysis of randomized controlled trials (RCTs). All eligible RCTs evaluating combination therapy for treating CHB were identified from nine electronic databases. A meta-analysis was performed in accordance with the Cochrane Systemic Review handbook. Eleven trials encompassing 1010 participants were included in this meta-analysis. It showed that at 12 and ≥ 96 weeks of therapy, the combination of ETV and IFN was not better than ETV in improving the undetectable HBV DNA (12 weeks: RR=1.12, 95% CI=0.88-1.42; ≥ 96 weeks: RR = 0.64, 95% CI=0.21-1.98, respectively) and HBeAg seroconversion rates (12 weeks: RR=1.35, 95% CI=0.60-3.04; ≥ 96 weeks: RR=1.36, 95% CI=0.75-2.64, respectively). But at 48 weeks of therapy and approximately 2 years of follow up, combination therapy was superior to ETV in improving the undetectable HBV DNA (48 weeks: RR=1.46, 95% CI=1.13-1.90; follow up: RR=2.20, 95% CI=1.26-3.81, respectively) and HBeAg seroconversion rates (48 weeks: RR=1.82, 95% CI=1.44-2.30; follow up: RR=1.92, 95% CI=1.19-3.11, respectively). When compared to IFN group, at 24 and 48 weeks of therapy, combination group showed a greater undetectable HBV DNA (24 weeks: RR=2.14, 95% CI=1.59-2.89; 48 weeks: RR=2.28, 95% CI=1.54-3.37, respectively) and ALT normalization rate (24 weeks: RR=1.56, 95% CI= 1.24-1.96; 48 weeks: RR=1.55, 95% CI = 1.16-2.07, respectively). At 48 weeks of therapy, combination group achieved a greater HBeAg seroconversion rate than IFN (48 weeks: RR=1.58, 95% CI=1.24-2.00). No significant differences were observed in the side effects of the three therapies. So we can conclude that ETV and IFN combination therapy is more effective than ETV or IFN mono-therapy in CHB treatment. ETV, IFN, and the combination of the two are safe in CHB treatment.

## Introduction

Liver disease associated with persistent hepatitis B virus (HBV) infection represents a major health problem with global impact. Approximately 2 billion people have been infected with HBV at one point, and over 350 million people suffer from chronic hepatitis B (CHB) worldwide [1]. The progression of HBV-related liver disease to cirrhosis, liver failure and hepatocellular carcinoma (HCC) is estimated to result in 0.5–1.2 million annual deaths [2]. Antiviral therapy is an effective way of preventing disease progression and even reversing liver fibrosis and cirrhosis [3–6]. The currently available treatments for CHB include two kinds of therapeutic agents: nucleoside and nucleotide analogues (NAs) and interferon (IFN) [7]. The NAs include lamivudine (LAM), telbivudine (LDT), entecavir (ETV), emtricitabine (FTC), adefovir dipivoxil (ADV) and tenofovir (TDF) [8]. IFN is divided into conventional IFN and pegylated IFN. The major advantages of the NAs are their high tolerability, effective suppression of HBV DNA replication and a high rate of on-treatment response. However, the drawbacks of NAs are also noteworthy; patients suffer from an indefinite treatment duration and drug resistance triggered by long-term therapy. In contrast, IFN is an immunomodulatory drug with a low rate of resistance, a finite course of treatment and potential long-term post-treatment responses. However, responses to IFN are only attained in a minority of patients, and severe adverse reactions make IFN poorly tolerated [9–11].

Currently, mono-therapy approaches with NAs or IFN cannot produce satisfactory antiviral effects. One theoretically viable strategy is the combination of NAs and IFN. The Japanese Guidelines for the treatment of HBV recommend that young HBeAg-negative patients with high HBV DNA level be treated sequentially with ETV followed by IFN as a first-line therapy [12]. At present, a combination therapy of NAs and IFN is not recommended in the guidelines proposed by the Asian-Pacific Association (updated in 2012) [13], the American Association (updated in 2009) [4] or the European Association for the Study of the Liver (updated in July 2012) [14]. Until now, the efficacy and safety of combination therapy has not been evaluated [15–16]. Recently, some systematic reviews have examined the combination of IFN and LAM or ADV, but the results are controversial [17–18]. Marcellin and colleagues have investigated LDT in combination with IFN, and concluded that for increased risk of peripheral neuropathy, combination therapy of the two should not be used [19].

ETV and TDF are similarly effective and safe in CHB treatment [20]. Both of them are more potent than LAM and ADV in suppressing HBV DNA and have a lower rate of resistance. However, TDF is more expensive than ETV. The effectiveness and safety of ETV and IFN combination therapy for CHB is uncertain. Several recent RCTs showed that a combination of ETV and IFN was superior to mono-therapy; however, other reports claimed that mono-therapies and combination therapy had similar results [21–31]. Because the sample sizes of the present RCTs are small and the consequences of each are incompatible, a more definitive conclusion is elusive. Since, HBeAg-positive CHB is characterized by high levels of HBV DNA, high risks of complications, high relapse rates, and a more pronounced need for efficacious therapy [13,32–33]. We conducted this meta-analysis to evaluate the efficacy and safety of ETV and IFN combination therapy in HBeAg-positive patients and to ultimately provide evidence for clinical decisions.

## Materials and Methods

### Literature search strategy

This systematic search was conducted independently by two researchers (Qiao-Ling Xie and Ling-Hong Wu). We searched Pubmed/Medline, the Cochrane Central Register of Controlled Trials, the Cochrane Database of Systematic Review databases, EMBASE, The Wiley Online library, Web of Science, The Chinese Journal of Science and Technology of VIP, The China National Knowledge Infrastructure (CNKI), and The Wanfang database for relevant literature. The latest article was published in October 2014. The publication language of each RCT was not restricted. The search strategy was based on a combination of the key words “hepatitis B or HBV or CHB,” “entecavir or ETV,” and “interferon or interferons or IFN”. We also searched reference lists and relevant reviews for additional articles.

### Inclusion and exclusion criteria

The articles included in this meta-analysis met the following inclusion criteria: 1) they were RCTs; 2) all patients infected with HBV had the following clinical indicators: HBsAg and HBeAg in the serum for more than 6 months, HBV DNA levels ≥ 10^5^ copies/ml and ALT levels > 2 times the upper normal limit; 3) the patients received initial treatment; 4) the studies compared the combination of ETV and IFN to ETV or IFN mono-therapy. Studies meeting the following exclusion criteria were excluded in this study: 1) non-RCTs; 2) co-infection with hepatitis A, C, D, or E, cytomegalovirus, or HIV; 3) anti-viral therapy was not performed initially; 4) patients had liver cirrhosis, liver failure, HCC, or other liver related complications caused by autoimmune diseases, drugs or alcoholism.

### Efficacy measures

Efficacy was evaluated based on the following criteria: undetectable HBV DNA: HBV DNA levels < 1,000 copies/ml; ALT normalization: ALT levels < 40 IU/ml; HBeAg seroconversion: HBeAg loss and occurrence of HBeAb. Drug safety was evaluated according to side effects, laboratory abnormalities, hepatitis flares, or death.

### Data extraction

Data extraction was carried out by two reviewers independently (Qiao-Ling Xie and Lin-Lin Fu). We recorded the following for each study: 1) trial characteristics (the first author’s name, published year, country of study, sum of each group, and quality of RCT); 2) patient characteristics (mean age, ethnicity of patients); 3) the details of each regimen (i.e., the antiviral drug used and treatment duration); and 4) observation time and outcomes. We contacted the authors of the eligible publications that had inadequate information; if effective data were still not obtained, those papers were excluded. All the data were reviewed to eliminate duplicate reports of the same trial.

### Assessment for risk of bias in the included studies

Methodological quality was defined as the confidence that the design and the report of the RCT would restrict bias in the comparison of the interventions [34]. According to empirical evidence [35–37], the methodological quality of the trials was assessed based on sequence generation, allocation concealment, blinding (of participants, personnel, and outcome assessors), incomplete outcome data, selective outcome reporting, and other sources of bias. The risk of each bias was defined (Table 1). We also used the Jadad scale to evaluate the quality of the RCTs [38]. Discrepancies were resolved by discussion with a third person (Ying Zhu).

**Table 1:** The definition of the risk of each bias.

Components	Sequence generation	Allocation concealment	Blinding	Incomplete outcomes	Selective reporting	Other bias
Low risk of bias	The method used is right (i.e., computer generated random numbers, table of random numbers).	The method used (i.e., central allocation) is unlikely to induce biases on the final observed effect.	Blinding was performed adequately, or the outcome measurement is not likely to be effected by lack of blinding.	The numbers and reasons for dropouts and withdrawals in all intervention groups were described or if it was specified that there were no dropouts or withdrawals.	Pre-defined, or clinically relevant and reasonably expected outcomes are reported on.	The trial appears to be free of other sources of bias.
Unclear risk of bias	The trial is described as randomized, but the method of sequence generation is not specified.	There is inadequate information to assess whether the method used is likely to induce biases.	There is inadequate information to assess whether the method used is likely to induce bias on the estimate of effect.	The report gave the impression that there had been no dropouts or withdrawals, but this was not stated in detail.	It is unclear whether data on these outcomes were recorded or not.	There is inadequate information to assess whether other sources of bias are present.
High risk of bias	The method used is improper and likely to introduce confounding.	The method used is likely to induce biases.	There is no blinding or the outcome measurement is likely to be effected by lack of blinding.	The number or reasons for dropouts and withdrawals were not described.	Not all of the trial’s pre-specified primary outcomes have been reported or similar.	There are other factors in the trial that could put it at risk of bias (i.e., lack of sample size or power calculation).

### Statistical analysis

This meta-analysis was conducted in accordance with the Preferred Reporting Items for Systematic Reviews and Meta-analysis (PRISMA) statement [39]. STATE V 12.0 (Intercooled state software 12.0) was used for the data analysis. All P values were two-tailed with a significance level of 0.05. For prospective studies and dichotomous data, we used relative risk (RR) as an effect measure and reported its 95% CI. This meta-analysis was performed using a random-effects model or fixed-effects model based on significant heterogeneity. Heterogeneity was evaluated via the I^2^ and P values. The fixed-effects method was used when the value of I^2^ was < 50% or when 50% < the value of I^2^ < 60%, but P > 0.1. The random-effects method was used when the value of I^2^ was > 60% or when 50% < the value of I^2^ < 60%, but P < 0.1 [40–41]. Sensitivity analysis was carried out to estimate the stability of the results. Begg’s rank correlation test and Egger’s linear regression test were used to assess publication biases.

## Results

### Study selection and baseline characteristics

**Fig 1:**
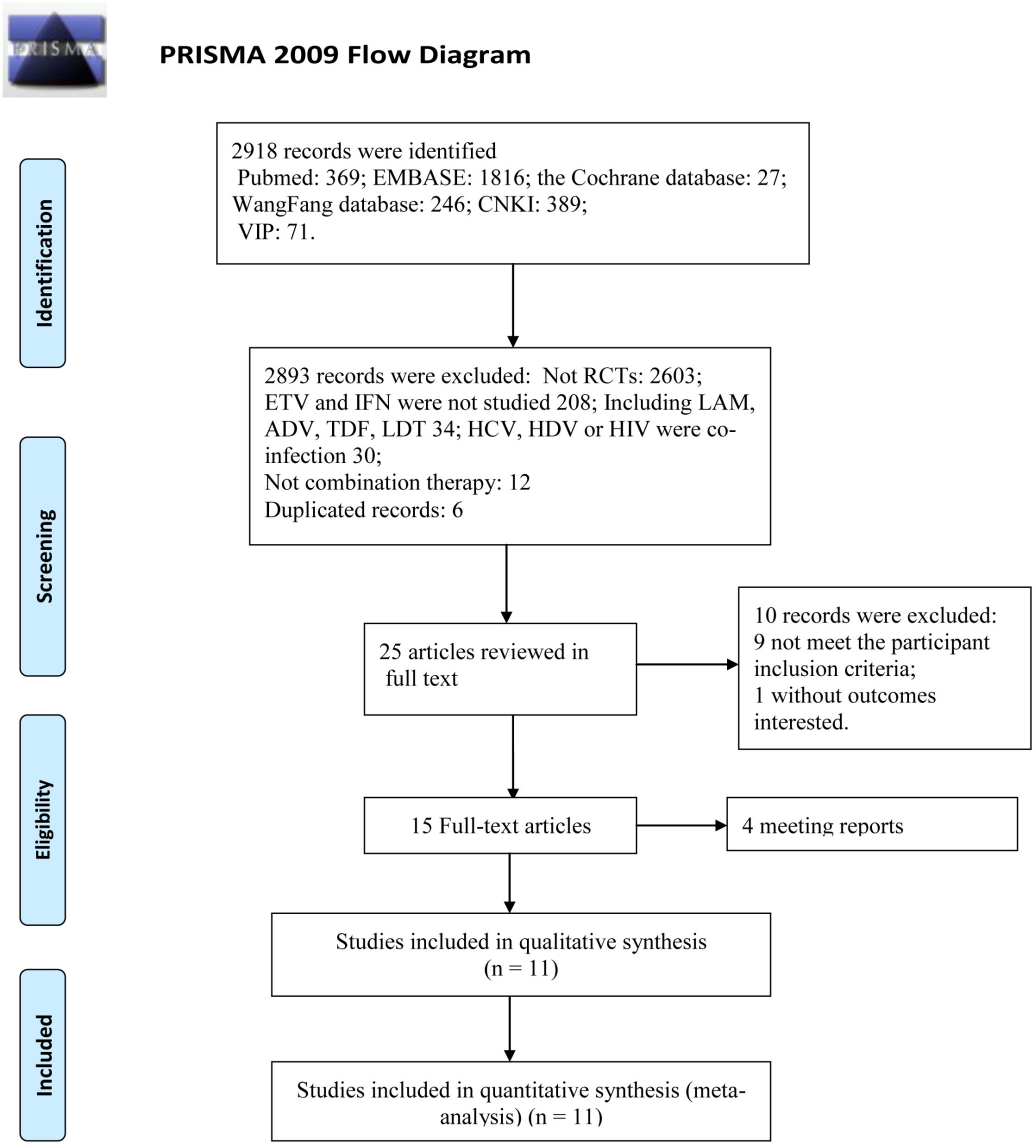
Flow Chart of Randomized Controlled Trials that were Evaluated. *From*: Moher D, Liberati A, Tetzlaff J, Altman DG, The PRISMA Group (2009). *P*referred *R*eporting *I*terns for *S*ystematic Reviews and *M*eta-*A*nalyses: The PRISMA Statement. PLoS Med 6(6): e1000097. doi:10.1371/journal.pmed1000097 For more information, visit www.prisma-statement.org.

We initially identified 2918 papers. By evaluating the titles and abstracts, those that were not RCTs, were duplicated, not involved with ETV and IFN, studied other NAs (e.g., LAM, ADV, LDT or TDF), were co-infection with other viruses, and ETV and IFN not used jointly were excluded, leaving 25 studies. By reviewing the full texts of these articles, finally, 11 trials (7 in Chinese and 4 in English) [21–31] were included in this meta-analysis (Fig 1). These studies include a total of 1010 patients: 439 were treated with combination therapy, 300 were treated with ETV mono-therapy, and 271 were treated with IFN mono-therapy. All studies reported the baseline characteristics of the 2 groups in detail. There were no significant differences in gender, age or duration of treatment between the two groups in these papers (Table 2).

**Table 2:** Main characteristics of studies included.

Num	Trial	Yr	Location	Ethnicity	Sample size	Mean age	Regimen	Observation	Outcomes	Jadad
					ETV/IFN/ETV+IFN	ETV/IFN/ETV+IFN Mean ± SD (yrs)	ETV/IFN/ETV+IFN	Time(wk)		
1	Li [21]	2012	China	Asian	51/43/ 44	43.9±12.8	ETV48wk/ɑ-IFN48wk/(ETV + ɑ-IFN)48wk	12,24,48	A,B,C,D	2
2	Mao[22]	2012	China	Asian	40/40/ 40	44.8±5.30, 43.2±6.8,4 7±5.9	ETV52wk/IFNɑ-1b52wk/ETV24wk+(IFNɑ-1b + ETV)4wk+IFNɑ-1b24wk	52,F	A,B,C,	5
3	Zeng [23]	2013	China	Asian	20/20/20	31.0±6.8, 30.9±4.6,30.8±6.5	ETV96wk/Peg-IFN48wk/(ETV + Peg-IFN)12wk+Peg-IFN36wk	4,12,24	A,B,C,D	3
4	Cui[24]	2013	China	Asian	36/36/36	41.7±5.9	ETV48wk/IFNɑ-2b48wk/ETV24wk+ (ETV + IFNɑ-2b)4wk+IFNɑ-2b20wk	48	A,B,C,D	5
5	Fan[25]	2012	China	Asian	00/40/40	48.9±0.4, 49.7±0.6	IFNɑ-2b48wk/ETV12wk+IFNɑ-2b36wk	12,24,48	A,B,C	2
6	L.B[26]	2013	Italy	White	00/20/ 20	39.0±7.5, 33.5±7.5	Peg-IFN48wk/ETV12wk+(ETV + Peg-IFN)12wk+Peg-IFN36wk	48,F	A,B,C	2
7	Xie[27]	2014	China	Asian	00/72/73	29.5±8.1, 29.2±6.9	peg-IFN48wk/peg-IFN13wk+(peg-IFN +ETV)24wk+peg-IFN11wk	48,F	A,B,C,D	5
8	Wei[28]	2012	China	Asian	12/00/ 13	35.4±11.0	ETV48wk/ETV24wk+(ETV + Peg-IFN)24wk	48	A,B,C,D	3
9	Chen [29]	2013	China	Asian	32/00/33	36.7±8.1, 38.4±11.6	ETV48wk/(ETV + IFNɑ-1b)48wk	12,24,48	A,B,C,D	5
10	Chen-C [30]	2012	Taiwan	Asian	19/00/35	25.6±43.5	ETV(72–96)wk/(ETV + Peg-IFN)24wk+Peg-IFN24wk	24,96	A,B,C,D	2
11	Brouwer [31]	2014	Global	Asian/White/Other	90/00/ 85	31±9, 32±10	ETV96wk/ETV24wk+(ETV + Peg-IFN)24 wk + ETV(24–48)wk	48,72,96,F	A,B,C,D	5

Note: ETV, entecavir; IFN, interferon; Peg-IFN, peginterferon ɑ-2a; SD, standard deviation; A, undetectable HBV-DNA; B, ALT normalization; C, HBeAg seroconvertion; D, adverse events; F, follow up; wk, weeks;

### Risk of bias of studies included

**Fig 2:**
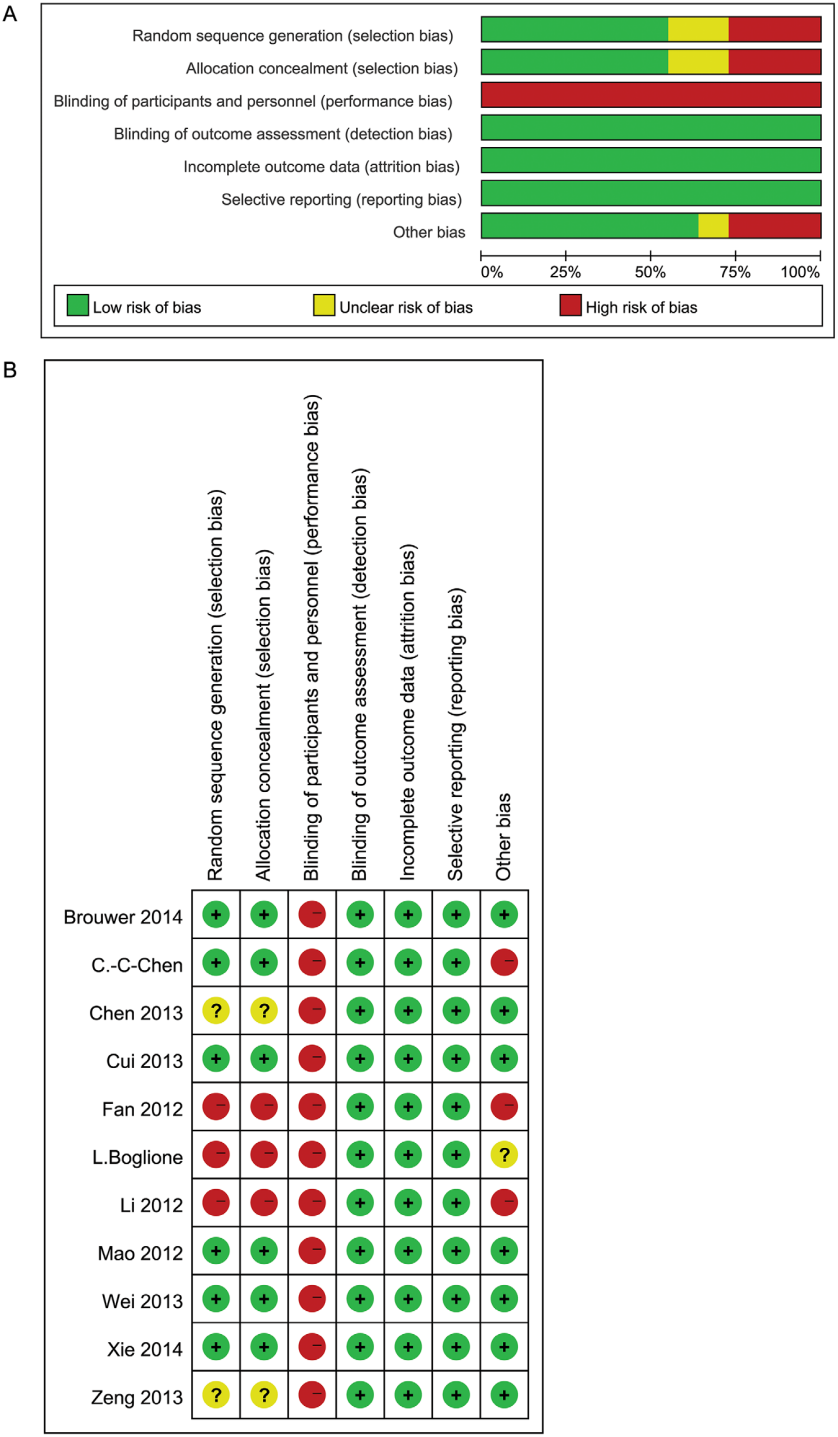
Risk of Bias in the Studies that were included in this Meta-analysis. (A) Review judgments of authors on each methodological quality item presented as percentages across all studies. (B) Review judgments of authors on each methodological quality item for each included study.

Eleven eligible studies were RCTs [21–31]. Five studies received Jadad scores of 5, and the others received scores of 2 or 3 (Table 2). 7 trials reported the randomization performed in the study, 5 of which described the method of randomization in detail [22,24,27,29,31]. The randomization methods in the other 4 studies were not appropriate [21,25–26,30]. All trials were open-labelled, but we do not expect this to have caused bias. The primary outcomes assessed (i.e., undetectable HBV DNA, ALT normalization, and HBeAg seroconversion) were unlikely to have been affected by knowledge of the intervention. All trials reported sample size calculations and complete data, and none of the patients dropped out of the trial. Four papers reported the results of approximately 2 years of follow up [22,26–27,31]. All trials reported at least one interested outcome parameters. All trials were likely to be free of selective reporting (Fig 2A and 2B).

Combination therapy with ETV and IFN was used in the trial group. ETV or IFN treatment was used in the control group, so we performed this meta-analysis to compare the differences between combination therapy and ETV or IFN mono-therapy.

### Undetectable HBV DNA

**Fig 3:**
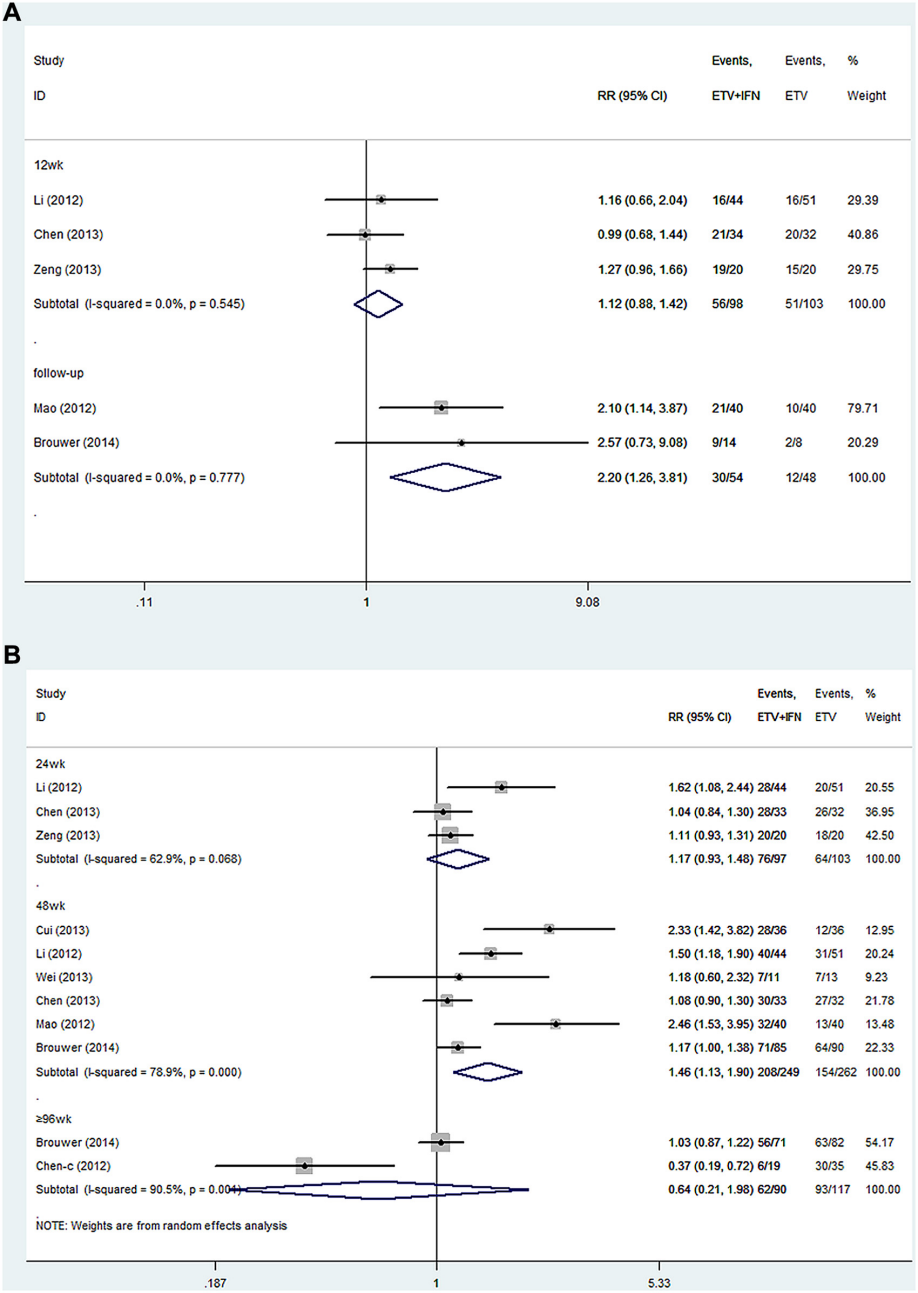
Forest Plot for Undetectable HBV DNA when ETV was used as the Control Group. (A) Forest plot for undetectable HBV DNA based on a fixed-effects model. (B) Forest plot for undetectable HBV DNA based on a random-effects model.

**Fig 4:**
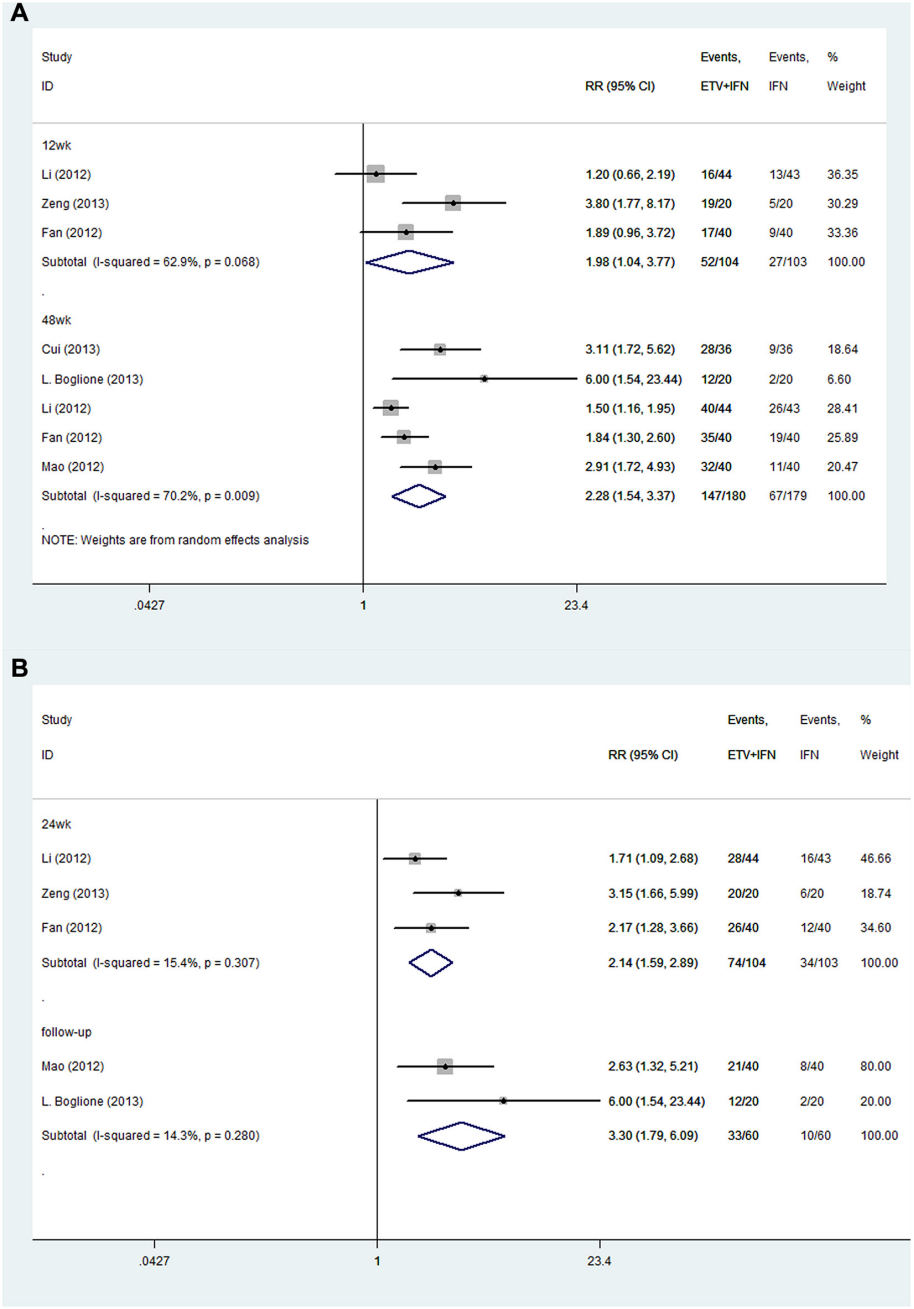
Forest Plot for Undetectable HBV DNA when IFN was used as the Control Group. (A) Forest plot for undetectable HBV DNA based on a random-effects model. (B) Forest plot for undetectable HBV DNA based on a fixed-effects model.

1. **ETV+IFN vs. ETV.** All the trials reported the rate of undetectable HBV DNA [21–24,28–31]. Because no significant heterogeneity was found across the studies at 12 weeks of treatment and approximately 2 years of follow up, we chose a fixed-effects model (12 weeks: I^2^ = 0.0%, P = 0.545; follow up: I^2^ = 0.0%, P = 0.771, respectively). Significant heterogeneity existed among studies at 24, 48 and ≥ 96 weeks of therapy, so we chose a random-effects model (24 weeks: I^2^ = 62.9%, P = 0.068; 48 weeks: I^2^ = 78.9%, P = 0.000; ≥ 96 weeks: I^2^ = 90.5%, P = 0.004, respectively). At 12, 24 and ≥ 96 weeks of therapy, the rate of undetectable HBV DNA was similar between the two groups (12 weeks: RR = 1.12, 95% CI = 0.88–1.42; 24 week: RR = 1.17, 95% CI = 0.93–1.48; ≥ 96 weeks: RR = 0.64, 95% CI = 0.21–1.98, respectively). However, at 48 weeks of therapy and approximately 2 years of follow up, a greater undetectable HBV DNA rate was observed in the combination group compared to the ETV group (48 weeks: RR = 1.46, 95% CI = 1.13–1.90; follow up: RR = 2.20, 95% CI = 1.26–3.81, respectively; Fig 3A and 3B).
2. **ETV+IFN vs. IFN.** All trials reported the rate of undetectable HBV DNA [21–27]. No heterogeneity was observed across the studies at 24 weeks of treatment and approximately 2 years of follow up; therefore, we chose a fixed-effects model (24 weeks: I^2^ = 15.4%, P = 0.307; follow up: I^2^ = 14.3%, P = 0.280, respectively). Heterogeneity was detected across the studies at 12 and 48 weeks of treatment, so we chose a random-effects model (12 weeks: I^2^ = 62.9%, P = 0.068; 48 weeks: I^2^ = 70.2%, P = 0.009, respectively). At 12, 24 and 48 weeks of therapy and approximately 2 years of follow up, a greater rate of undetectable HBV DNA was observed in the combination therapy group compared to the IFN group (12 weeks: RR = 1.98, 95% CI = 1.04–3.77; 24 weeks: RR = 2.14, 95% CI = 1.59–2.89; 48 weeks: RR = 2.28, 95% CI = 1.54–3.37; follow up: RR = 3.30, 95% CI = 1.79–6.09; Fig 4A and 4B).

### ALT normalization

**Fig 5:**
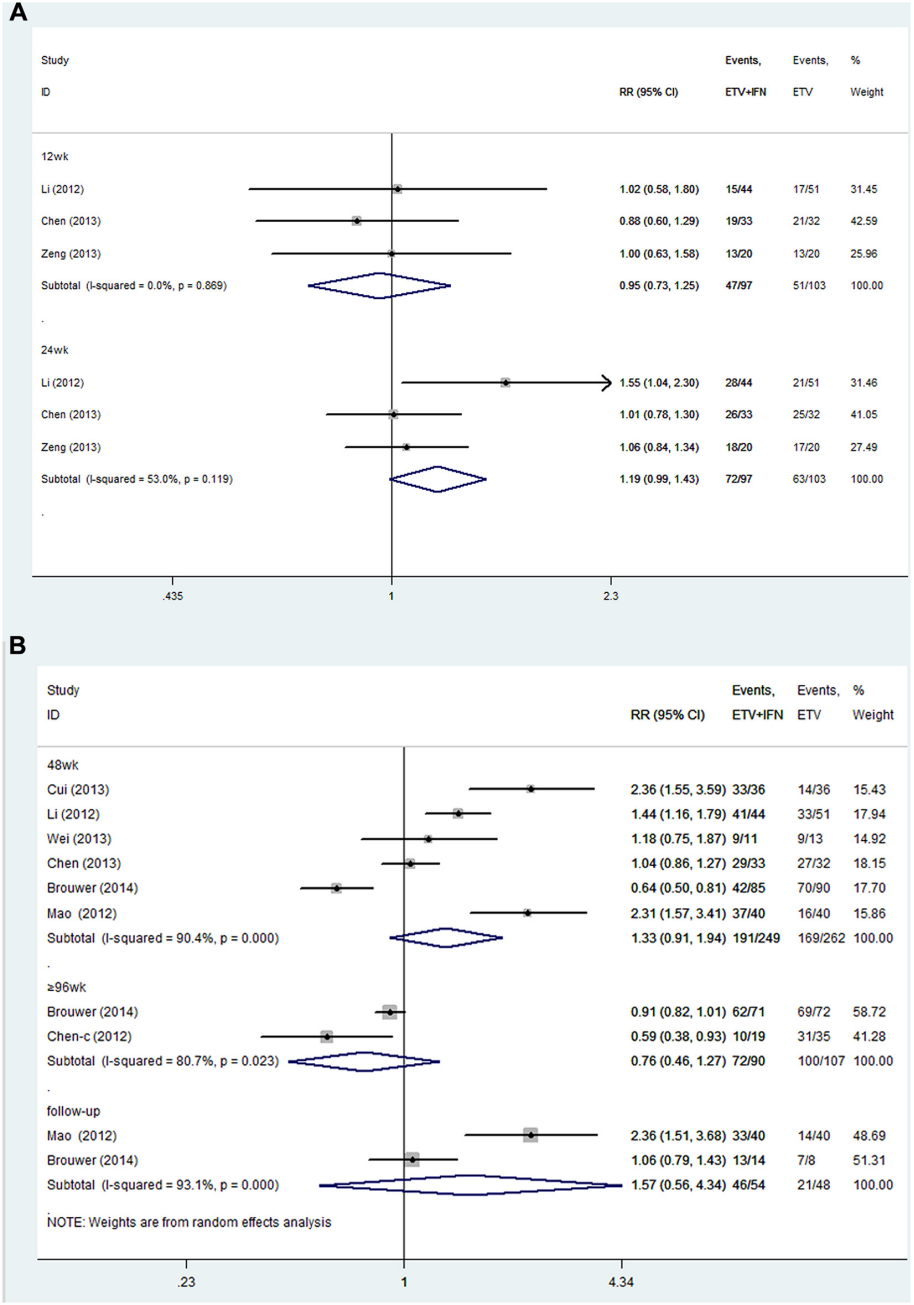
Forest Plot for ALT Normalization when ETV was used as the Control Group. (A) Forest plot for ALT normalization based on a random-effects model. (B) Forest plot for ALT normalization based on a fixed-effects model.

**Fig 6:**
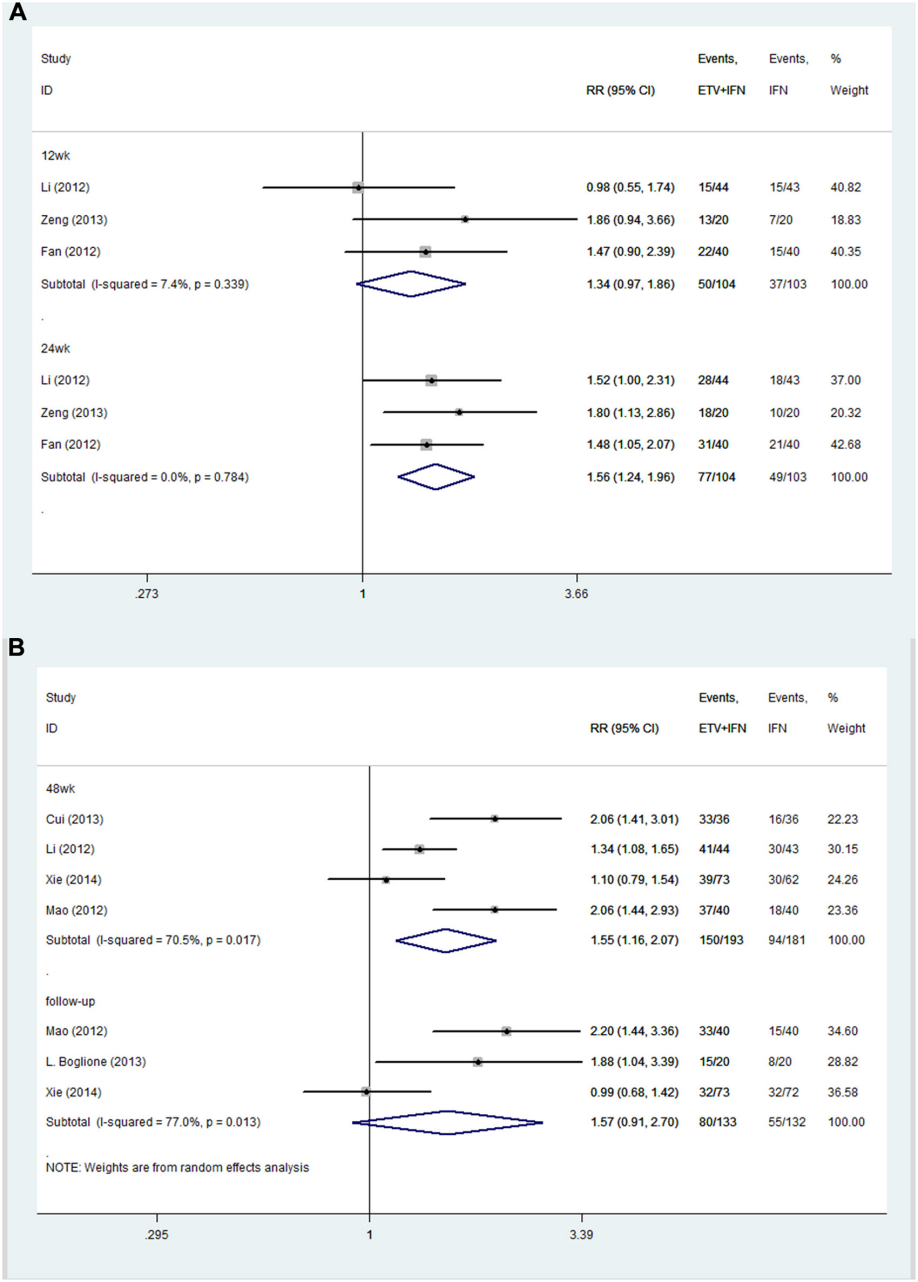
Forest Plot for ALT Normalization when IFN was used as the Control Group. (A) Forest plot for ALT normalization based on a random-effects model. (B) Forest plot for ALT normalization based on a fixed-effects model.

1. **ETV+IFN vs. ETV.** All trials reported the rate of ALT normalization [21–24,28–31]. Because no significant heterogeneity was detected across the studies at 12 and 24 weeks of treatment, we chose a fixed-effects model (12 weeks: I^2^ = 0.0%, P = 0.869; 24 weeks: I^2^ = 53%, P = 0.119, respectively). Significant heterogeneity existed among studies at 48 and ≥ 96 weeks of treatment and approximately 2 years of follow up, so we chose a random-effects model (48weeks: I^2^ = 90.4%, P = 0.000; ≥96 weeks: I^2^ = 80.7%, P = 0.023; follow up: I^2^ = 93.1%, P = 0.000, respectively). At 12, 24, 48 and ≥ 96 weeks of therapy and approximately 2 years of follow up, the rates of ALT normalization were similar between the two groups (12 weeks: RR = 0.95, 95% CI = 0.73–1.25; 24 weeks: RR = 1.19, 95% CI = 0.99–1.43; 48 weeks: RR = 1.33, 95% CI = 0.91–1.94; ≥ 96 weeks: RR = 0.76, 95% CI = 0.46–1.27; follow up: RR = 1.57, 95% CI = 0.56–4.34, respectively; Fig 5A and 5B).
2. **ETV+IFN vs. IFN.** All trials reported the rate of ALT normalization [21–27]. Because no significant heterogeneity was found across the studies at 12 and 24 weeks of treatment, we chose a fixed-effects model (12 weeks: I^2^ = 7.4%, P = 0.339; 24 weeks: I^2^ = 0.0%, P = 0.784, respectively). Heterogeneity was observed among studies at 48 weeks of treatment and approximately 2 years of follow-up; we therefore chose a random-effects model (48 weeks: I^2^ = 70.5%, P = 0.017; follow up: I^2^ = 77.0%, P = 0.013, respectively). The results showed that at 12 weeks and approximately 2 years of follow up, the rate of ALT normalization was similar between the two groups (12 weeks: RR = 1.34, 95% CI = 0.97–1.86; follow up: RR = 1.57, 95% CI = 0.91–2.70, respectively). However, at 24 and 48 weeks of therapy, combination therapy achieved higher ALT normalization rates than the IFN group (24 weeks: RR = 1.56, 95% CI = 1.24–1.96; 48 weeks: RR = 1.55, 95% CI = 1.16–2.07, respectively; Fig 6A and 6B).

### HBeAg seroconversion

**Fig 7:**
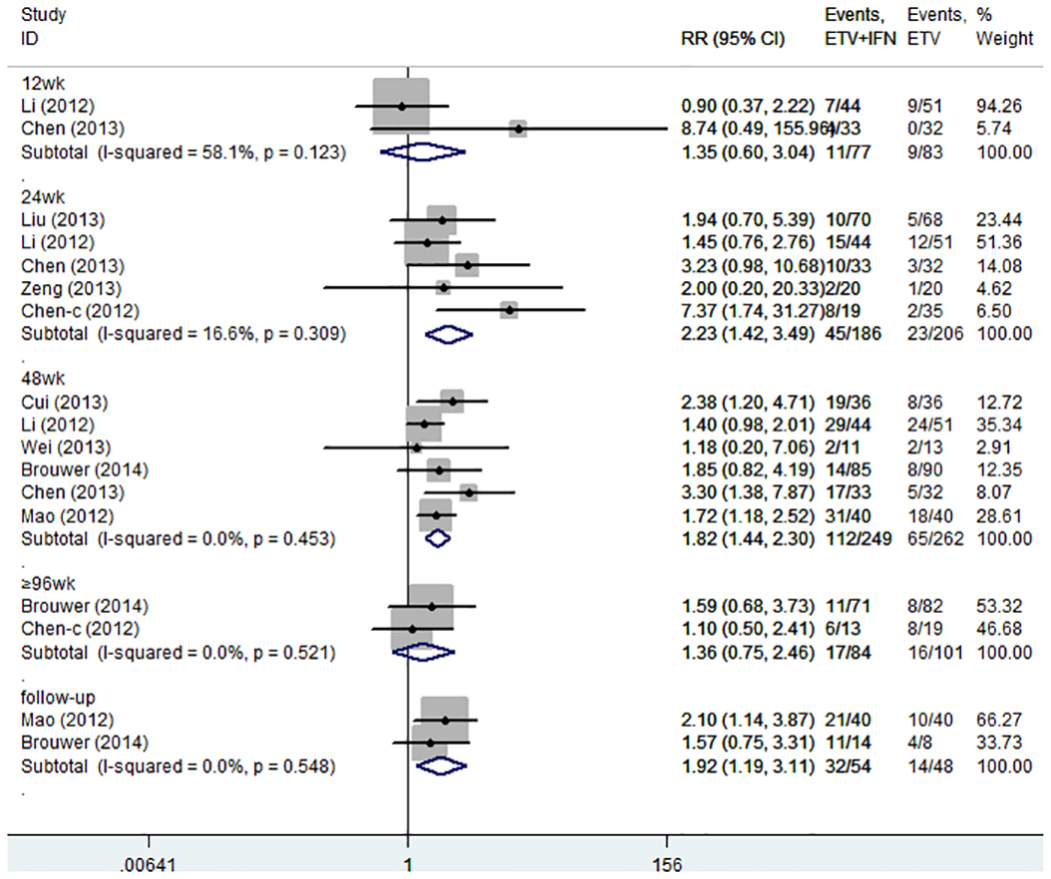
Forest Plot for HBeAg Seroconversion when ETV was used as the Control Group.

**Fig 8:**
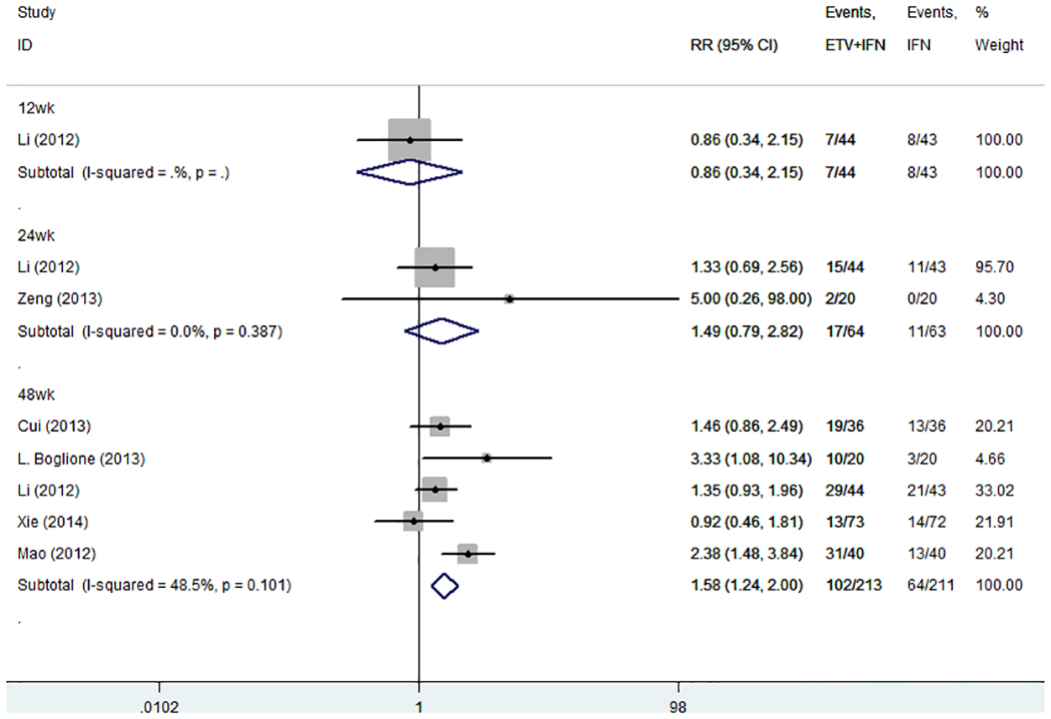
Forest Plot for HBeAg Seroconversion when IFN was used as the Control Group.

1. **ETV+IFN vs. ETV.** All trials reported the rate of HBeAg seroconversion [21–24,28–31]. No significant heterogeneity was observed; we therefore chose a fixed-effects model (12 weeks: I^2^ = 58.1%, P = 0.123; 24 weeks: I^2^ = 16.6%, P = 0.309; 48 weeks: I^2^ = 0.0%, P = 0453; ≥ 96 weeks: I^2^ = 0.0%, P = 0.521; follow up: I^2^ = 0.0%, P = 0.548, respectively). The results showed that at 12 and ≥ 96 weeks of therapy, the rate of HBeAg seroconversion was similar in the two groups (12 weeks: RR = 1.35, 95% CI = 0.60–3.04; ≥ 96 weeks: RR = 1.36, 95% CI = 0.75–2.46, respectively). However, at 24 and 48 weeks of therapy and approximately 2 years of follow up, the combination therapy group achieved greater HBeAg seroconversion rates than the ETV group (24 weeks: RR = 2.23, 95% CI = 1.42–3.49; 48 weeks: RR = 1.82, 95% CI = 1.44–2.30; follow up: RR = 1.92, 95% CI = 1.19–3.11, respectively; Fig 7).
2. **ETV+IFN vs. IFN.** Six trials reported the rates of HBeAg seroconversion [21–24,26–27]. Because no significant heterogeneity was found, we chose a fixed-effects model (12 weeks: I^2^ = .%, P = .; 24 weeks: I^2^ = 0.0%, P = 0.387; 48 weeks: I^2^ = 48.5%, P = 0.101, respectively) The results showed that at 12 and 24 weeks of therapy, the rate of HBeAg seroconversion was similar in the two groups (12 weeks: RR = 1.86, 95% CI = 0.34–2.15; 24 weeks: RR = 1.49, 95% CI = 0.79–2.82, respectively). However, at 48 weeks of therapy combination therapy achieved greater HBeAg seroconversion rates than the IFN group (48 weeks: RR = 1.58, 95% CI: 1.24–2.00; Fig 8).

### Adverse reactions

Eight trials reported adverse reactions [21,23–24,27–31]. Cui reported no significant differences in adverse events between the combination group and the IFN group (p> 0.05, 69.44%, 77.78%) [24]. Li Jin and Xie *et al*. reported that there were some common side effects including influenza-like illness, fatigue, gastrointestinal symptoms, weight loss, hair loss, myelosuppression and neuropsychiatric problems in both the combination group and IFN group [21,27]. Xie *et al*., Wei *et al*. and Chen C-C *et al*. observed hyperthyroidism in the combination group [27–28,30]. Wei *et al*. estimated that there were no differences in the rate of glomerular filtration decrease between the combination group and ETV group. Additionally, there were no severe hepatitis flares or decompensation reported. The mild side effects of combination therapy were tolerable and did not necessitate early termination or dose reduction.

### Sensitivity analysis

Some of the I^2^ heterogeneity values were large, so we performed sensitivity analysis with a random-effects model. Sensitivity analysis was performed for all models with high heterogeneity. This analysis showed that the pooled RRs were similar before and after removal of each trial, and no single trial significantly altered the pooled RRs. This suggests that these results are stable (Table 3).

**Table 3:** Sensitivity analysis for all outcomes with high heterogeneity.

Outcomes	Times	Study omitted	RR	95%CI
HBV DNA (A)	24 weeks	Li(2012)	1.08	0.95–1.24
		Chen(2013)	1.31	0.75–2.29
		Zeng(2013)	1.27	0.77–2.08
		Combined	1.17	0.93–1.48
	48 weeks	Cui (2013)	1.35	1.06–1.71
		Li(2012)	1.48	1.06–2.05
		Wei (2013)	1.50	1.13–2.00
		Chen (2013)	1.60	1.17–2.18
		Mao (2012)	1.33	1.06–1.65
		Brouwer (2014)	1.59	1.08–2.32
		Combined	1.46	1.13–1.90
	96 weeks	Brouwer(2014)	0.37	0.19–0.72
		Chen-c(2012)	1.03	0.87–1.22
		Combined	0.64	0.21–1.98
HBV DNA (B)	12 weeks	Li(2012)	2.62	1.32–5.18
		Zeng(2013)	1.47	0.94–2.30
		Fan(2012)	2.08	0.67–6.44
		Combined	1.98	1.04–3.77
	48 weeks	Cui(2013)	2.10	1.39–3.18
		L.Boglione(2013)	2.10	1.46–3.01
		Li(2012)	2.62	1.75–3.92
		Fan(2012)	2.61	1.42–4.77
		Mao(2012)	2.13	1.38–3.28
		Combined	2.28	1.54–3.37
ALT normalization (A)	48 weeks	Cui(2013)	1.19	0.81–1.75
		Li(2012)	1.31	0.81–2.11
		Wei(2013)	1.36	0.88–2.09
		Brouwer(2014)	1.55	1.09–2.20
		Mao(2012)	1.19	0.81–1.75
		Combined	1.33	0.91–1.94
	96 weeks	Brouwer(2014)	0.60	0.38–0.93
		Chen-C(2012)	0.91	0.82–1.00
		Combined	0.76	0.46–1.27
	follow up	Mao(2012)	1.06	0.79–1.43
		Brouwer(2014)	2.36	1.51–3.68
		Combined	1.57	0.56–4.34
ALT normalization (ALT)	48 weeks	Cui(2013)	1.43	1.05–1.96
		Li(2012)	1.66	1.09–2.53
		Xie(2014)	1.74	1.23–2.45
		Mao(2012)	1.42	1.05–1.94
		Combined	1.55	1.16–2.07
	follow up	Mao(2012)	1.30	0.70–2.43
		L.Boglione(2013)	1.46	0.67–3.21
		Xie(2014)	2.08	1.48–2.94
		Combined	1.57	0.91–2.70

Note: A, using ETV as a control group; B, using IFN as a control group; RR, Relative Risk; CI, Confidence interval.

### Publication bias

We performed Begg’s test and Egger’s test to evaluate the occurrence of publication bias. The results were listed in Table 4. There was no evidence of publication biases except for the outcome parameter of undetectable HBV-DNA (ETV+IFN vs. IFN). Thus, we cautiously concluded that the biases in this meta-analysis were not obvious.

**Table 4:** Publication bias for all outcomes included.

Model	Outcomes	Begg's Test		Egger's test		
		Z	Pr>z	T	Pt	95% CI
A	HBV DNA	1.04	0.30	1.40	0.18	-0.66, 3.11
B	HBV DNA	2.56	0.01	3.93	0.00	1.08, 3.87
A	ALT	0.77	0.44	1.72	0.11	-0.50, 4.50
B	ALT	0.43	0.67	0.92	0.38	-1.61, 3.91
A	HBeAg	1.52	0.13	1.74	0.10	-0.19, 1.84
B	HBeAg	-0.12	1.00	0.34	0.74	-2.26, 3.01

Note: A, ETV+IFN vs. ETV; B, ETV+IFN vs. IFN

## Discussion

HBV is a small, partially double-stranded DNA virus that belongs to the hepadnaviridae family [42]. The natural course of chronic HBV infection consists of four phases: immune tolerance, immune reactive, inactive carrier and reactivation phase [7, 14]. To date, the efficacy and safety of mono-therapy with IFN or NAs has been unsatisfactory [43]. The suboptimal outcomes of the current treatment options for CHB prompt the exploration of their use in combination to achieve synergistic efficacy and decreased mutagenicity [44]. In our study, we focused on the effectiveness and safety of ETV and IFN combination therapy.

This meta-analysis analyzed four outcome parameters: the undetectable HBV DNA rate, ALT normalization rate, HBeAg seroconversion rate and adverse reactions. The HBV DNA level is a primary marker for appraising the treatment responses of patients with CHB [45–46]. The early and sustained suppression of HBV DNA replication produces long-term virological, biochemical and serological responses [47]. HBeAg is a protein expressed by the pre-C gene. HBeAg seroconversion is a key point of treatment responses and is a necessary condition for halting drug therapy for HBeAg-positive patients [48].

When ETV mono-therapy was used as the control group, this meta-analysis estimated that combination therapy achieved greater HBV DNA undetectable rates (at 48 weeks of therapy) and HBeAg seroconversion rates (at 24 and 48 weeks of therapy) than the control group. However, at ≥ 96 weeks of therapy, we found that the rate of HBV DNA undetectable and HBeAg seroconversion was similar between the two groups. This suggested that, when compared with ETV, combination therapy achieved transient superiority; however, at the late stage of treatment, the efficacy of the two groups was similar. Maybe, IFN and ETV were combined transiently at an early stage of treatment in some trials. However, it seemed contradictory that significant differences were observed once again at 2 years of follow up. It is possible that only few outcomes were investigated at 2 years of follow up [22,31]; therefore, this result may not be reliable, and a much bigger sample size RCT is needed. We can only conclude that combination therapy can achieve a superior response than ETV mono-therapy at an early stage of treatment.

When IFN was used as the control group, this meta-analysis showed that at 48 weeks of therapy, combination therapy achieved greater virological, biochemical and serological response rates than IFN mono-therapy. There are several potential reasons for this result: 1) ETV may reinforce the antiviral effect in the combination group by suppressing HBV DNA replication; 2) A high HBV DNA load is related to an inefficient T cell response to HBV-related antigens [49]; and 3) It has been hypothesized that the inhibition of viral replication by ETV can decrease the HBV-related protein synthesis on the surface of hepatocytes, which may restore the immune response and contribute to the immunomodulatory activity of IFN for infected cells clearance. Boni and his colleagues have also reported that a decreased viral load induced by NAs therapy can lead to the subsequent restoration of CD8 and CD4 cellular immune responses against HBV [50–51]. We can therefore conclude that combination therapy consisting of ETV and IFN is more rapid and effective than IFN mono-therapy in HBeAg-positive CHB.

There were eight articles reported adverse effects. Side effects with ETV therapy have rarely been reported. When compared to IFN, the adverse reactions of the two groups were similar. The emergence of new and different adverse events was not observed in the combination group. Therefore, we can cautiously suggest that combination therapy is safe and tolerable, but long-term observation is needed.

Some limits merit consideration. First, the differences between conventional IFN and pegylated IFN were not further evaluated in subgroup analysis. Second, the differences between the initial combination therapy and sequential combination therapy were not further discussed in subgroup analysis because of the small number of relevant articles. Third, the quality of some of the included trials was not high because details about the methods of randomization, allocation, concealment, and blinding were unclear.

Our meta-analysis indicated that ETV and IFN combination therapy is more effective than ETV or IFN mono-therapy in HBeAg-positive CHB treatment. The combination of the two is also safe in the treatment of CHB. However, there are still some limits to combination therapy: first, combination therapy is very expensive; second, a definite duration for combination therapy is unclear; and third, it is uncertain that whether an initial combination therapy approach or a sequential therapy approach is more suitable. Therefore, studies with much larger sample sizes are needed to explore the advantages of combination therapy.

## Supporting Information

S1 Checklist PRISMA checklist. (DOC)

S1 Table Characteristics of studies excluded. (DOC)
