# Supplementary material for: The Efficacy and Safety of Entecavir and Interferon Combination Therapy for Chronic Hepatitis B Virus Infection: A Meta-Analysis
Source: PLoS One. 2015 Jul 30;10(7):e0132219. doi: 10.1371/journal.pone.0132219 (PMC4520608; doi:10.1371/journal.pone.0132219)
Supplement: S1 Checklist — (DOC) [file pone.0132219.s001.doc]

| **Section/topic** | **#** | **Checklist item** | **Reported on page #** |
| --- | --- | --- | --- |
| **TITLE** | | |  |
| Title | 1 | The Efficacy and Safety of Entecavir and Interferon Combination Therapy for Chronic Hepatitis B Virus Infection: A Meta-analysis | 1 |
| ABSTRACT | | |  |
| Structured summary | 2 | The objective of this study was to evaluate the effectiveness and safety of entecavir (ETV) and interferon (IFN) combination therapy in the treatment of chronic hepatitis B (CHB) mono-infection via a meta-analysis of randomized controlled trials (RCTs). All eligible RCTs evaluating combination therapy for treating CHB were identified from nine electronic databases. A meta-analysis was performed in accordance with the Cochrane Systemic Review handbook. Eleven trials encompassing 1010 participants were included in this meta-analysis. The result showed that at 12 and ≥ 96 weeks of therapy, combination of ETV and IFN was not better than ETV in improving the rates of undetectable HBV DNA (12 weeks: RR=1.12, 95%CI=0.88-1.42; ≥ 96 weeks: RR = 0.64, 95% CI=0.21-1.98, respectively) and HBeAg seroconversion(12 weeks: RR=1.35, 95% CI=0.60-3.04; ≥ 96 weeks: RR=1.36, 95% CI=0.75-2.64, respectively). But at 48 weeks of therapy and approximately 2 years of follow up , combination therapy was more effective than ETV in improving the undetectable HBV DNA (48 weeks: RR=1.46, 95% CI=1.13-1.90; follow up: RR=2.20, 95% CI=1.26-3.81, respectively) and HBeAg seroconversion rates (48 weeks: RR=1.82, 95% CI=1.44-2.30; follow up: RR=1.92, 95% CI=1.19-3.11, respectively). When compared to IFN group, at 24 and 48 weeks of therapy combination group showed a greater undetectable HBV DNA (24 weeks: RR=2.14, 95% CI=1.59-2.89; 48 weeks: RR=2.28, 95% CI=1.54-3.37, respectively) and ALT normalization rate (24 weeks: RR=1.56, 95% CI= 1.24-1.96; 48 weeks: RR=1.55, 95% CI = 1.16-2.07, respectively). At 48 weeks of therapy, combination group achieved a greater HBeAg seroconversion rate than IFN (48 weeks: RR=1.58, 95% CI=1.24-2.00). No significant differences were observed in the side effects of the three therapies. So we can conclude that ETV and IFN combination therapy was more effective than ETV or IFN mono-therapy in CHB treatment. ETV, IFN, and the combination of the two were safe in CHB treatment. | 2-3 |
| INTRODUCTION | | |  |
| Rationale | 3 | Liver disease associated with persistent hepatitis B virus (HBV) infection represents a major health problem with global impact. Approximately 350 million people suffer from chronic hepatitis B (CHB) worldwide [1]. The progression of HBV-related liver disease to cirrhosis, liver failure and hepatocellular carcinoma (HCC) is estimated to result in 0.5-1.2 million annual deaths [2]. Antiviral therapy is an effective way of preventing disease progression and even reversing liver fibrosis and cirrhosis [3-6]. The currently available treatments for CHB include two kinds of therapeutic agents: nucleoside (nucleotide) analogues (NAs) and interferon (IFN) [7]. The nucleoside analogues include lamivudine (LAM), telbivudine (LDT), entecavir (ETV) and emtricitabine (FTC), and the nucleotide analogues include adefovir dipivoxil (ADV) and tenofovir (TDF) [8]. IFN is divided into conventional IFN and pegylated IFN. The major advantages of the NAs are their high tolerability, effective suppression of HBV DNA replication and a high rate of on-treatment response to therapy. However, the drawbacks of NAs are also noteworthy: an indefinite treatment duration and drug resistance triggered by long-term therapy. In contrast, IFN is an immunomodulatory drug with a low rate of resistance, a finite course of treatment and potential long-term post-treatment responses. However, responses to IFN are only attained in a minority of patients, and severe adverse reactions make IFN poorly tolerated [9-11]. | 3-4 |
| Objectives | 4 | ETV and TDF are similarly effective and safe in CHB treatment [20], and both of them are more potent than LAM and ADV in suppressing HBV DNA with a lower rate of resistance. However, TDF is more expensive than ETV. The effectiveness and safety of ETV and IFN combination therapy for CHB is uncertain. Several recent RCTs, such as the studies of Li Jin and Brouwer WP, showed that a combination of ETV and IFN in HBeAg-positive patients was superior to mono-therapy; however, other reports claimed that mono-therapies and combination therapy had similar results [21-31]. Because the sample sizes of the present RCTs were small and the consequences of each were incompatible, a more definitive conclusion was elusive. Since, HBeAg-positive CHB is characterized by high HBV DNA load, high risks of complications, high relapse rate after the cessation of treatment, and a more pronounced need for efficacious therapy [13,32-33]. We conducted this meta-analysis to compare the efficacy and safety of ETV and IFN combination therapy in HBeAg-positive patients and to ultimately provide evidence for clinical decisions. | 4-5 |
| METHODS | | |  |
| Protocol and registration | 5 | Indicate if a prospective protocol exists, if and where it can be accessed (e.g., Web address), and, if available, provide registration information including registration number. | N/A |
| Eligibility criteria | 6 | The articles included in this meta-analysis met the following inclusion criteria: 1) they were RCTs; 2) all patients infected with HBV had the following clinical indicators: HBsAg and HBeAg in the serum for more than 6 months, HBV DNA levels ≥ 105 copies/ml and ALT levels >2 times the upper normal limit; 3) the patients received initial treatment; 4) intervention therapy of ETV and IFN combination therapy. | 5-6 |
| Information sources | 7 | We searched Pubmed/Medline, the Cochrane Central Register of Controlled Trials, the Cochrane Database of Systematic Review databases, EMBASE, The Wiley Online library, Web of Science, The Chinese Journal of Science and Technology of VIP, The China National Knowledge Infrastructure (CNKI), and The Wanfang database for relevant literature. The latest article was published in Oct, 2014. | Materials and Methods  6 |
| Search | 8 | The search strategy was based on a combination of key words “hepatitis B” or “hbv” or “CHB”, “entecavir” or “ETV” and “interferon” or “interferons” or “IFN”. We also searched reference lists and relevant reviews for additional articles. | Materials and Methods  6 |
| Study selection | 9 | State the process for selecting studies (i.e., screening, eligibility, included in systematic review, and, if applicable, included in the meta-analysis). | Materials and Methods  6 |
| Data collection process | 10 | Data extraction was carried out by two reviewers independently (Qiao-Ling Xie and Lin-Lin Fu). | Materials and Methods  Page 6 |
| Data items | 11 | We recorded the following for each study: 1) trial characteristics (the first author’s name, published year, country of study, sum of each group, and quality of RCT); 2) patient characteristics (mean age, ethnicity of patients); 3) the details of each regimen (i.e., the antiviral drug used and treatment duration); and 4) observation time and outcomes. | Materials and Methods  6 |
| Risk of bias in individual studies | 12 | Methodological quality was defined as the confidence that the design and the report of the RCT would restrict bias in the comparison of the interventions [34]. According to empirical evidence [35-37], the methodological quality of the trials was assessed based on sequence generation, allocation concealment, blinding (of participants, personnel, and outcome assessors), incomplete outcome data, selective outcome reporting, and other sources of bias. We also used the Jadad scale to evaluate the quality of the RCTs | Materials and Methods  7 |
| Summary measures | 13 | Forest plots were used to estimate the the efficacy of antiviral including HBV DNA undetectability, ALT normalization and HBeAg seroconversion All P values were two-sided and a P value of less than 0.05 was considered to be statistically significant. | Materials and Methods  9 |
| Synthesis of results | 14 | Forest plots were used to estimate the efficacy of antiviral. Meta analysis was performed using a random-effect model or fixed-effect model basing on the significant heterogeneity. Heterogeneity was evaluated by the value of I-squared and P. The fixed-effect method was used in this situation: the value of I-squared <50% or 50 %< the value of I-squared <60% but P>0.1. The random-effect method was used when the value of I-squared >60%or 50% < the value of I-squared <60% but P<0.1[29-30]. | Materials and Methods  9 |

| **Section/topic** | **#** | **Checklist item** | **Reported on page #** |
| --- | --- | --- | --- |
| Risk of bias across studies | 15 | Sensitivity analysis was made to estimate result stability. The Begg’s rank correlation test and Egger’s linear regression test were used to assess publication biases. | Materials and Methods  9 |
| Additional analyses | 16 | No. | Materials and Methods |
| **RESULTS** | | |  |
| Study selection | 17 | We initially identified 2918 papers by evaluating the titles and abstracts, and those that were not RCTs, were duplicated, not involved with ETV and IFN, studied other NAs (e.g., LAM, ADV, LDT or TDF), were co-infection with other viruses, and ETV and IFN not used jointly were excluded, leaving 25 studies. By reviewing the full texts of these articles, finally, 11 trials (7 in Chinese and 4 in English) [21-31] were included in this meta-analysis (Fig. 1). | Results  9-10 |
| Study characteristics | 18 | Finally, 11 trials (7 in Chinese and 4 in English) [21-31] were included in this meta-analysis (Fig. 1). These studies include a total of 1010 patients: 439 were treated with combination therapy, 300 were treated with ETV mono-therapy, and 271 were treated with IFN mono-therapy. All studies reported the baseline characteristics of the 2 groups in detail. There were no significant differences in gender, age or duration of treatment between the two groups in these papers (Table 2). | Results  9-10 |
| Risk of bias within studies | 19 | The details of risk of bias of included RCTs were on Figure 2.A and B, additionally, Table 2. | Result  9-10 |
| Results of individual studies | 20 |  | Table 2 |
| Synthesis of results | 21 | We used a random-effect model or fixed-effect model basing on the significant heterogeneity  ETV+IFN vs ETV: At 12 weeks, 24 weeks and ≥96 weeks of therapy, the rate of undetectable HBV DNA was similar between the two groups (12 weeks: RR=1.12, 95% CI 0.88-1.42; 24 week: RR=1.17, 95% CI 0.93-1.48; ≥96 weeks: RR=0.64, 95% CI 0.21-1.98, respectively). However, at 48 weeks of therapy and approximately 2 years of follow up, a greater undetectable HBV DNA rate was observed in the combination group compared to the ETV group (48 weeks: RR1.46, 95% CI 1.13-1.90; follow up: RR= 2.20, 95% CI 1.26-3.81, respectively; Figs. 3A and 3B).At 12, 24, 48 and ≥ 96 weeks of therapy and approximately 2 years follow up, the rates of ALT normalization were similar between the two groups (12 weeks: RR=0.95, 95% CI: 0.73-1.25; 24 weeks: RR=1.19, 95% CI: 0.99-1.43; 48 weeks: RR=1.33, 95% CI: 0.91-1.94; ≥ 96 weeks: RR=0.76, 95% CI: 0.46-1.27; follow up: RR =1.57, 95% CI: 0.56-4.34, respectively; Figs. 5A and 5B).The results showed that at 12 weeks and ≥96 weeks of therapy, the rate of HBeAg seroconversion was similar in the two groups (12 weeks: RR=1.35, 95% CI: 0.60-3.04; ≥96 weeks: RR=1.36, 95% CI: 0.75-2.46). However, at 24 and 48 weeks of therapy and approximately 2 years of follow up, the combination therapy group achieved greater HBeAg seroconversion rates than the ETV group (24 weeks: RR=2.23, 95% CI: 1.42-3.49; 48 weeks: RR=1.82, 95% CI: 1.44-2.30; follow up: RR=1.92, 95% CI: 1.19-3.11, respectively; Fig. 7).  ETV+IFN vs IFN:At 12 weeks, 24 weeks and 48 weeks of therapy and approximately 2 years follow up, a greater rate of undetectable HBV DNA was observed in the combination therapy group compared to the IFN group (12 weeks: RR=1.98, 95% CI: 1.04-3.77; 24 weeks: RR=2.14, 95% CI: 1.59-2.89; 48 weeks: RR=2.28, 95% CI: 1.54-3.37; follow up: RR=3.30, 95% CI: 1.79-6.09; Figs. 4A and 4B).The results showed that at 12 weeks and approximately 2 years of follow up the rate of ALT normalization was similar between the two groups (12 weeks: RR=1.34, 95% CI 0.97-1.86; follow up: RR=1.57, 95% CI: 0.91-2.70, respectively) . However, at 24 and 48 weeks of therapy, combination therapy achieved higher ALT normalization rates than the IFN group (24 weeks: RR=1.56, 95% CI: 1.24-1.96; 48 weeks: RR=1.55, 95% CI: 1.16-2.07, respectively; Figs. 6A and 6B).The results showed that at 12 and 24 weeks of therapy, the rate of HBeAg seroconversion was similar in the two groups (RR=1.86, 95% CI: 0.34-2.15; RR=1.49, 95% CI: 0.79-2.82, repectively). However, at 48 weeks of therapy combination therapy achieved greater HBeAg seroconversion rates than the IFN group (48 weeks: RR=1.58, 95% CI: 1.24-2.00; Fig. 8). | Results  13-18 |
| Risk of bias across studies | 22 | Some of the I^2^ heterogeneity values were large, so we performed sensitivity analysis with a random-effects model. Sensitivity analysis was performed for undetectable HBV DNA rate at 48 weeks of treatment (using ETV as a control group); this analysis showed that the pooled RRs were similar before and after the removal of each study in turn, and no single trial significantly altered the pooled RRs. It suggests that the results are stable (Table 3).  We performed Begg’s test and Egger’s test to evaluate the occurrence of publication bias. The outcomes are listed in Table 4. There was no evidence of publication biases except for the outcome parameter of undetectable HBV-DNA (ETV+IFN vs. IFN). Thus, we cautiously concluded that the biases in this meta-analysis were not obvious. | Results  18-19 |
| Additional analysis | 23 | No. | Results |
| **DISCUSSION** | | |  |
| Summary of evidence | 24 | Summarize the main findings including the strength of evidence for each main outcome; consider their relevance to key groups (e.g., healthcare providers, users, and policy makers). | Discussion  19-22 |
| Limitations | 25 | First, when IFN was considered as the control group (including ɑ-IFN, IFN ɑ-1b, IFN ɑ-2b and Peg-IFNɑ-2a), the regimen of each treatment was stated as being matched to standard treatment in the included articles. Second, combination therapy in this meta-analysis consisted of both initial combination therapies and sequential combination therapies, however, the difference between the two approaches were not further discussed in subgroup analysis because of the small number of relevant articles. Third, the quality of some included trials was not high because details about the methods of randomization, allocation, concealment, and blinding were not clear. | Discussion  22 |
| Conclusions | 26 | Our meta-analysis indicates that combination therapy is efficacious and safe. The subgroup analysis of 2 years of follow up estimates that combination therapy can produce better sustained responses than mono-therapy. | Conclusions  22-23 |
| **FUNDING** | | |  |
| Funding | 27 | The National Science Foundation of China, No.81273925. |  |
