# Supplementary material for: The Efficacy and Safety of Entecavir and Interferon Combination Therapy for Chronic Hepatitis B Virus Infection: A Meta-Analysis
Source: PLoS One. 2015 Jul 30;10(7):e0132219. doi: 10.1371/journal.pone.0132219 (PMC4520608; doi:10.1371/journal.pone.0132219)
Supplement: S1 Table — (DOC) [file pone.0132219.s002.doc]

Table 4. Characteristics of excluded studies. (ordered by year of study )

| Study | Reason for exclusion |
| --- | --- |
| Moon  2011 | RCTs, but it is a meeting report. |
| Kim  2011 | RCTs; However, it is a meeting report. |
| Milian  2012 | RCTs; However, it is a meeting report. And interested outcome parameters could not be attained. |
| Liu  2013 | RCTs; However, it is a meeting report. |
| Chen  2013 | RCTs; However it did not meet participant inclusion criteria. All patients had been treated with ETV for at least 96weeks with HBV DNA ≤500 copies/ml |
| Zhou  2013 | RCTs; However, it did not meet participant inclusion criteria. All patients were HBV –DNA<10^5^copies/ml; |
| Zhang  2013 | RCTs; However, it did not meet participant inclusion criteria. All patients had been treated with IFN at first. |
| Hu  2014 | RCTs; However, it did not meet participant inclusion criteria. The HBV DNA level was not clear. And interested outcome parameters could not be attained. |
| Ayikuli  2014 | RCTs; However, it did not meet participant inclusion criteria. Treatment of part patients were not performed first time. |
| Yuan  2014 | RCTs; However, it did not meet participant inclusion criteria. All patients were treated with ETV at first. |
| Na  2014 | RCTs; However, it did not meet participant inclusion criteria. Patients were negative HBeAg. |
| Zhang  2014 | RCTs; However, it did not meet participant inclusion criteria. All patients were treated with IFN at first. |
| Chi c  2014 | RCTs; However, it did not meet participant inclusion criteria. All patients were treated with ETV or TDF at least 12 months. Then, Patients were randomized to 48 weeks PEG-IFN addition, or 48 weeks of continued NAs mono-therapy. |
| Zhang  2014 | RCTs; However, it did not meet participant inclusion criteria. Treatment of part patients were not performed first time. |
